# Myocardial deformation in children post cardiac surgery, a cross-sectional prospective study

**DOI:** 10.1186/s43044-024-00578-z

**Published:** 2024-11-18

**Authors:** Mohammad Ahmad Hassan, Ali Al-Akhfash, Yasser Bhat, Abdullah Alqwaiee, Mohammed Abdulrashed, Saad Saleh Almarshud, Abdulrahman Almesned

**Affiliations:** 1 PSCCQ: Prince Sultan Cardiac Center in Qassim, Buraidah, Saudi Arabia; 2 Pediatric department, Faculty of Medicine, Sohag University, Sohag, Egypt

**Keywords:** Congenital heart disease, Cardiac surgery, Cardiac function, Myocardial strain

## Abstract

**Background:**

Myocardial deformation by speckle tracking echocardiography provides valuable information on the left ventricular function. The study aims to assess myocardial deformation in terms of left ventricular strain as an indicator of myocardial function in children after cardiac surgery at outpatient follow-up visits.

**Methods:**

The study design was a prospective observational cross-sectional study that included pediatric patients after biventricular cardiac surgery during the postoperative follow-up visits in the outpatient department. In addition to conventional echocardiographic examination, two-dimensional speckle tracking echocardiography was done to evaluate myocardial deformation in terms of left ventricular strain. Echocardiographic measurements were done offline and were compared to published reference normal values for age. Study subjects were divided according to age at follow-up into four groups (1 month–1 year, 1–2 years, 2–5 years, and 5–11 years).

**Results:**

Over ten months, 100 patients (64 males and 36 females) were included in the study. The median age was 30.8 months (IQR 12.8–65.3 months), the median weight was 11.7 kg (IQR 8–17 kg) and the median duration after surgery was 7.3 months (IQR 3.2–30.8 months). Longitudinal strain values were significantly (*p* < 0.001) lower than reference values for different age groups. Global circumferential strain showed no significant difference from the reference values. The duration after surgery had a statistically significant effect on longitudinal strain values, with improvement of the strain values with increasing intervals after surgery.

**Conclusion:**

Using myocardial deformation method to evaluate cardiac function may detect underlying cardiac function abnormalities even with normal traditional functional parameters, which could have implications for patient management and follow-up.

## Background

Myocardial function is an important prognostic determinant of cardiopulmonary pathologies in children [1, 2]. The left ventricular (LV) myocardium has a complex architecture and consists of circumferential fibers in the mid-wall layer and longitudinal fibers in the endocardial and epicardial layers [3]. This results in inhomogeneous and complex contraction patterns, as the myofiber orientation changes continuously from the right-handed helix in the subendocardium to the left-handed helix in the subepicardium [4]. LV deformation comprises radial thickening, circumferential shortening, and longitudinal shortening; myocardial strain describes this deformation. Specifically, two-dimensional speckle tracking echocardiography (2DSTE) is a method for myocardial strain measurement used to estimate deformation measures and quantitatively characterize LV function in children [5–7].

Traditional parameters for myocardial function assessment, such as shortening fraction by motion-mode, ejection fraction by two-dimensional brightness-mode imaging, and myocardial velocities by pulsed-wave tissue Doppler imaging, provide valuable data on global cardiac function but have significant limitations. These limitations include high inter- and intra-observer variability, load dependency, and angle of insonation dependency. Myocardial deformation by 2DSTE partially overcomes these limitations and provides data on myocardial systolic strain and strain rates [6, 8]. It has been used to assess myocardial strain and strain rate in various pediatric cardiac and noncardiac diseases that can affect myocardial functions [2, 9]. In children post cardiac surgery, 2DSTE has been used to study the myocardial function either in the early postoperative period, [10–13] or at late follow-up visits after surgery [14, 15]. The study aims to assess the myocardial deformation in terms of left ventricular strain as an indicator of myocardial function using 2DSTE in children after cardiac surgery at outpatient follow-up visits at different intervals after surgery.

## Methods

The study was conducted at the Pediatric Cardiology Unit outpatient department at Prince Sultan Cardiac Center-Qassim in Saudi Arabia from October 2022 to July 2023. The prospective observational cross-sectional study included pediatric patients less than 14 years of age during outpatient follow-up visits after successful biventricular cardiac surgery with no significant residual lesions. Patients who had undergone palliative surgeries, such as pulmonary artery banding, single ventricle repair, or surgery for coronary abnormalities, were excluded. Additionally, children who had LV dysfunction (defined as an ejection fraction < 55%) before or after surgery were not considered for the study. Furthermore, newborns who were younger than one month of age were not included. Informed written consent was obtained from the study subjects’ caregivers before enrollment. The Qassim Regional Research Ethics Committee approved the study under reference number 607/44/10173. In accordance with established protocols, all patient information was treated with the utmost confidentiality, and no identifying details were disclosed at any stage of the process.

**Fig. 1:**
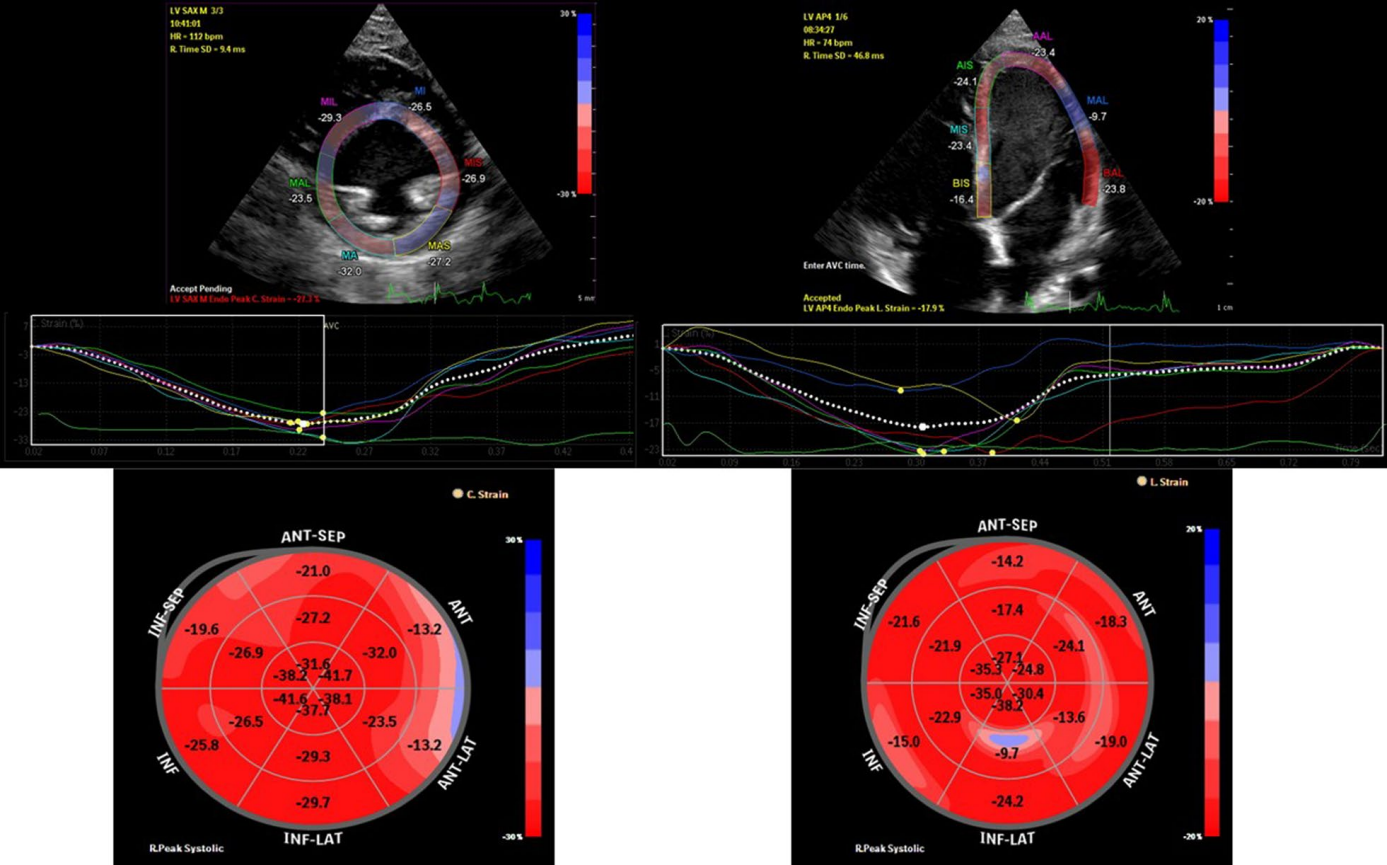
Myocardial strain measurements. The upper panel shows automated tracking of the region of interest at the short-axis mid-ventricular level on the left and the apical four-chamber level on the right. The lower panel shows corresponding 18-segment strain measurements

The children underwent a routine transthoracic echocardiographic assessment, including measurement of LV myocardial function by M-mode and two-dimensional brightness-mode imaging (Simpson’s method). In Addition, grayscale images of the LV optimized for speckle tracking echocardiographic analysis were acquired over three consecutive cardiac cycles in the three apical standard views (4, 3, and 2 chambers views) and the short-axis views at the apical, mid-ventricular, and basal levels. The Gray-scale images were analyzed offline by a single investigator (M.H). Regional myocardial function was assessed by longitudinal and circumferential strain, expressing percent change in segment length from end-diastole. End-diastole was defined as the peak of the *R* wave in the QRS complex on an electrocardiogram. The endocardial border was drawn manually. After automated tracking of the region of interest, manual correction was performed if necessary. The longitudinal regional function was measured in all six wall regions (anteroseptal, anterior, lateral, posterior, inferior, and inferoseptal) of the LV, with each wall automatically subdivided into three segments (18 segments model) (Fig. 1). Longitudinal and circumferential peak systolic strain and for all segments were obtained and averaged to express global LV longitudinal and circumferential strain. Transthoracic echocardiographic examination and offline analysis were done using the EPIQ 7 ultrasound system (Phillips Medical Systems, USA, Andover).

The statistical analysis was performed using SPSS Software (v. 26.0). The normal distribution was verified by the Shapiro–Wilk test. For continuous variables, we have presented the data as means and standard deviations, or as median and interquartile ranges. Continuous variables were compared using a one-sample t-test and paired t-test when appropriate. Independent samples Anova or Kruskal Wallis test were used when appropriate to compare the study groups. Linear regression analysis was used for correlation. Inter- and intraobserver variabilities were assessed by the intraclass correlation coefficient. The null hypothesis was rejected if *p* value < 0.05.

## Results

All patients were asymptomatic and in sinus rhythm at resting ECG. We studied 100 patients (demographics are shown in Table 1) with 108 measurements (some patients were studied more than once at successive follow-up visits). Thirteen patients had trisomy 21, three patients were dysmorphic with unknown syndrome, and 84 patients were nonsyndromic. The most frequent surgery was ventricular septal defect (VSD) closure (30%), followed by atrial septal defect (ASD) closure (15%) and tetralogy of Fallot (TOF) repair (14%) (Table 2). According to the postoperative follow-up period, there were 27 measurements during the first three months, 20 from three to six months, 21 from six to 12 months, and 40 more than one year after surgery.

**Table 1:** Demographics of study subjects (*N* = 100)

Variable	Median (IQR)/mean ± SD
Age (months)	30.8 (12.8–65.3)
Gender	64 male, 36 female
Weight (kg)	11.7 (8–17)
BAS (m^2)^	0.54 (0.4–0.74)
HR (bpm)	110 ± 24
Duration after surgery (months)	7.3 (3.2–30.8)
Age at surgery (months)	9.5 (5.5–37.6)
Weight at surgery (kg)	7 (5–12.6)

*N* number of patients; *kg* kilogram; *m*^*2*^ square meter; *bpm* beats per min, *IQR* interquartile range; *SD* standard deviation

**Table 2:** The distribution of surgical procedures (*N* = 100)

Surgical procedure	Number/percent %
VSD closure	30
ASD closure	15
TOF repair	14
AVSD repair	13
Aortic arch repair	12
RVOT obstruction repair	5
SAM resection	3
TAPVC repair	3
Others	5

*N* number of studied patients; *%* percent; *VSD* ventricular septal defect, *ASD* atrial septal defect, *TOF* tetralogy of fallot, *AVSD* atrioventricular septal defect, *RVOT* right ventricular outflow tract, *SAM* subaortic membrane, others included one patient for each of: tricuspid valve repair, total anomalous pulmonary venous connection repair, aortopulmonary window repair, arterial switch, and cortriatriatum repair

Study subjects were divided according to age at follow-up into four groups (1 month–1 year, 1–2 years, 2–5 years, and 5–11 years). Global longitudinal and circumferential strain for all patients were − 19.5 ± 3.6 with a frame rate of 75.7 ± 11 and − 26.6 ± 5.6 with a frame rate of 77.6 ± 10.6, respectively. The strain values at each age group were compared to the corresponding published reference values (Table 3) [16]. Longitudinal strain values: global, apical four chambers, three chambers, and two chambers were significantly (*p* < 0.001) lower than the reference values for different age groups. However, Circumferential strain values showed either non-significant differences or statistically significant higher values compared to the reference values. There was no significant effect of gender on either longitudinal or circumferential strain values.

**Table 3:** Longitudinal strain (LS) and circumferential strain (CS) mean ± SD in the study groups compared to reference values

	1 month–1 year (*n*. 25)			1–2 years (*n*. 27)			2–5 years (*n*. 27)			5–11 years (n.29)		
	Mean ± SD	Ref.	*P*	Mean ± SD	Ref.	*P*	Mean ± SD	Ref.	*P*	Mean ± SD	Ref.	*P*
LS 4 chamber	− 19.4 ± 4.2	− 26 ± 2.4	< 0.001	− 21 ± 4.3	− 26 ± 2.4	< 0.001	− 20.3 ± 3.8	− 25.7 ± 2.6	< 0.001	− 19.6 ± 4	− 25.1 ± 2.6	<0 .001
LS 3 chamber	− 20 ± 5.6	− 25.4 ± 3.3	< 0.001	− 18.7 ± 4.3	− 25.4 ± 3.3	<0 .001	− 19.4 ± 5	− 24.1 ± 3.1	<0 .001	− 17 ± 7.5	− 23.8 ± 2.9	<0 .001
LS 2 chamber	− 20 ± 5.7	− 26.7 ± 2.8	< 0.001	− 19.5 ± 4.5	− 26.7 ± 2.8	<0 .001	− 19.2 ± 7.9	− 25.6 ± 2.7	<0 .001	− 19.7 ± 5.5	− 25.4 ± 2.7	<0 .001
Global LS	− 19.7 ± 4.3	− 26 ± 2.3	< 0.001	− 19.6 ± 3.4	− 26 ± 2.3	<0 .001	− 20.2 ± 2.9	− 25 ± 2.2	<0 .001	− 19.3 ± 4	− 24.7 ± 2.3	<0 .001
CS basal	− 23 ± 9.1	− 22.1 ± 4.8	0.63	− 20 ± 12.1	− 22.1 ± 4.8	0.38	− 23 ± 6	21.3 ± 4.4	0.14	− 21.6 ± 5.6	− 22 ± 4.6	0.73
CS midventricular	− 27 ± 7.4	− 23.4 ± 6.3	0.03	− 26 ± 5.6	− 23.4 ± 6.3	0.04	− 26.6 ± 6.3	− 23.5 ± 4.7	0.051	− 25.6 ± 5.5	− 24.8 ± 4.8	0.47
CS apical	− 33.3 ± 9.4	− 28 ± 8.5	0.01	− 34.6 ± 6.4	− 28 ± 8.5	<0 .001	− 28.8 ± 10.2	− 25.7 ± 5.9	0.12	− 31 ± 5.8	− 26.9 ± 6.7	0.001
Global CS	− 27 ± 7.3	− 24.6 ± 4.2	0.12	− 27.5 ± 4	− 24.6 ± 4.2	0.01	− 26.5 ± 6	− 23.3 ± 4.3	0.01	− 25.8 ± 4.6	− 24.5 ± 4.5	0.15

*LS* longitudinal strain, *CS* circumferential strain. *n*. is the number of measurements in each age group

All measurements were done by a single investigator (MH). Intraobserver reliability of the strain measurements was assessed by blinded repeated measurements of a subset of stored data performed by the same investigator (MH). Another set of measurements was compared with blinded measurements by another investigator (AA) to assess inter-observer reliability. Intra- and inter-observer reliability demonstrated good agreement (Intraclass correlation coefficient 0.91 and 0.8, respectively, *p* < 0.001).

**Fig. 2:**
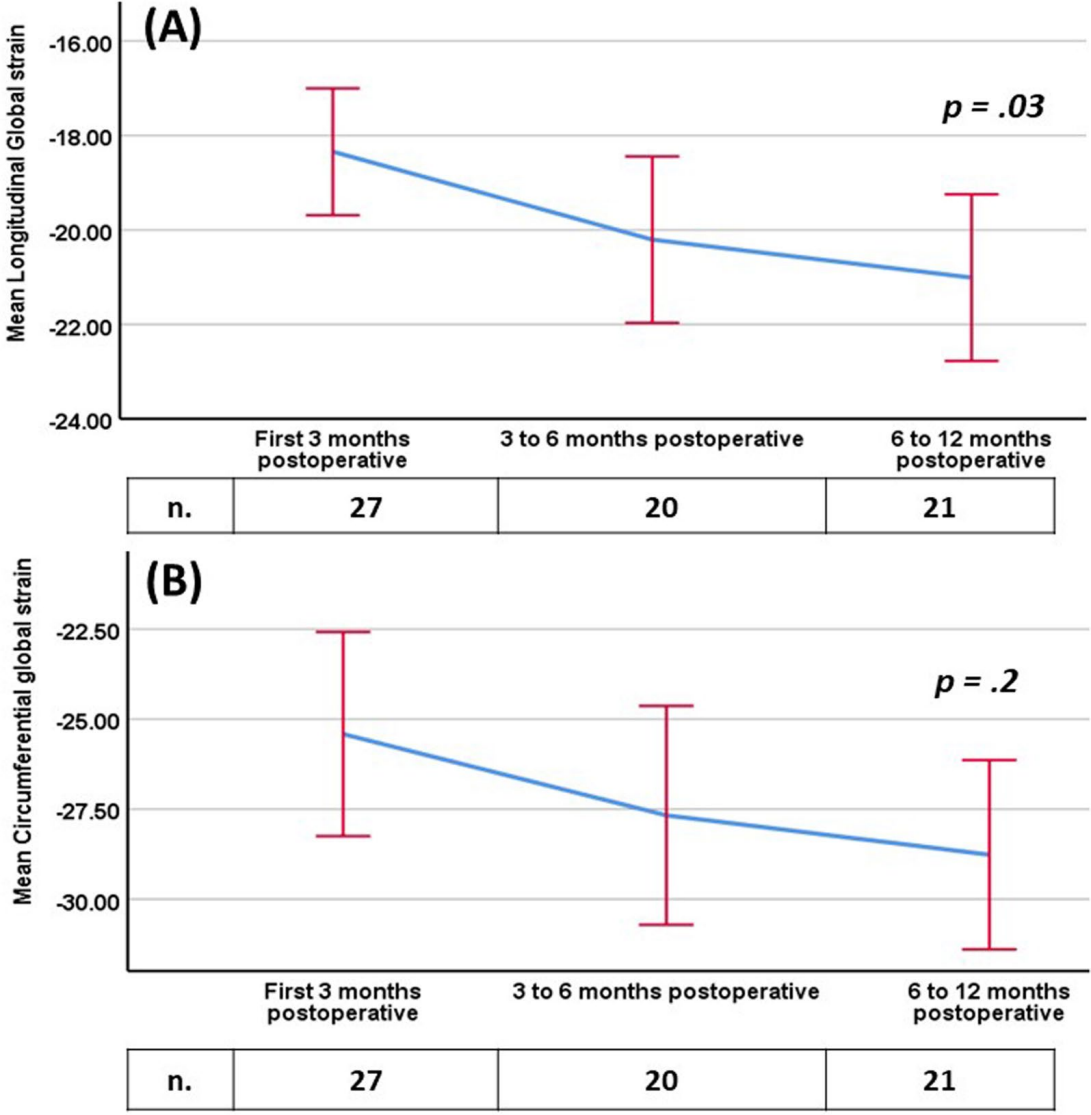
Mean global longitudinal (**A**) and circumferential (**B**) strain ± standard deviation at postoperative intervals during the first year after surgery

Regarding the timing of the study post-cardiac surgery, there was a significant increase in the global longitudinal strain, *F* (2.63) = 3.42, *p* = 0.03 with increasing duration after cardiac surgery, especially during the first year of follow-up (Fig. 2). In 40 cases we had only a follow-up after one year of cardiac surgery, in whom there was age and weight heterogeneity. In this group of patients, no timing trend of the longitudinal and circumferential strain was traced.

The mean EF measured by M-mode and Simpson method were 66.8 ± 6.6% and 63.5 ± 5.3%. M-mode derived EF before surgery was 63.4 5% and was significantly lower than EF measured at follow up after surgery (*p* = 0.003). There was no significant correlation between global strain values and operative age, operative weight, cardiopulmonary bypass time, cross-clamp time, postoperative mechanical ventilation hours, ICU stay, or hospital stay. We have studied children with 13 different surgical interventions. These groups of patients had significantly different ages and weight at the time of the study. Therefore, their strain values were not suitable to be compared against each other. However, the pairwise comparison of the mean percent of normal (87 ± 11%, 74 ± 13%, 75 ± 12.5%, and 77 ± 12.5% after surgical repair of ASD, VSD, TOF, and AVSD, respectively) was not statistically significant.

## Discussion

Conventional echocardiographic measurements of ejection fraction and fractional shortening have limitations in evaluating myocardial function and detecting early stages of decreasing LV systolic function. They are highly operator-dependent and heavily dependent on image quality, geometrical variables, and image orientation. Speckle-tracking echocardiography provides a promising emerging tool for early detection of LV systolic dysfunction that is not hindered by these limitations [6, 8].

This study measured longitudinal and circumferential LV strain using 2DSTE in children after cardiac surgery. Study subjects were divided according to age at follow-up into four groups (1 month–1 year, 1–2 years, 2–5 years, and 5–11 years). The data were compared to published reference normal values for each age group. [16] Longitudinal strain values were significantly lower than reference values for different age groups. Global circumferential strain values showed either nonsignificant differences or statistically significant higher values compared to the reference values.

In twenty-two children at least nine years of age after arterial switch operation for D-TGA. The global LV longitudinal strain of − 18.3 ± 1.3%, was significantly lower than healthy controls, and a global circumferential of − 26 ± 2.5%, was not different from the healthy control group. There was no significant correlation between strain values and operative variables (cardiopulmonary bypass time and cross-clamp time). The decrease in longitudinal strain in this patient group could indicate subclinical myocardial dysfunction [17].

At more than one year after correction of TOF, patients aged 8–18 years [18], 5–25 years [19], 1.5–16 years [20], and 18.7 ± 6 years [21] showed a decrease in LV strain compared to healthy controls, and it decreased with increasing age. The LV longitudinal strain was reported to be in the range of − 19.07 ± 2.1819 shortly (on the 8th day) after TOF repair, which was significantly lower than normal controls [12].

Perdreau et al. studied thirty-three patients with a mean age of 4.2 years before and immediately after different cardiac surgeries: ASD, VSD, AVSD, TOF, TGA, LVOTO, and valvular insufficiency. They found significant differences in longitudinal and radial strain but no difference in circumferential strain between preoperative and postoperative measurements [22]. Similar results were reported in 25 children with a mean age of 9.4 years studied preoperatively, one week, and one month postoperatively [10].

The findings in the current study agree with the abovementioned studies, with either near-term or long-term postoperative results showing global longitudinal strain significantly lower than normal values either measured preoperatively or compared to normal controls. Our findings are consistent with previous studies that have reported no significant differences in circumferential strain [17, 22]. The decrease in longitudinal strain values may be due to the selective hypoperfusion of longitudinal fibers, resulting in severe ischemia [23].

The present study presents a significant improvement in longitudinal strain in patients following cardiac surgery within the first year. However, the study findings indicate that the longitudinal strain values remain significantly lower than normal while having normal left ventricle ejection fraction by conventional echocardiography. This outcome emphasizes the importance of continued monitoring and managing patients after cardiac surgery to optimize their recovery and long-term outcomes. According to data reported by Van der Ende et al., the strain values recovered after surgical intervention of aortic stenosis or aortic coarctation. However, the values never reached normal even after a mean follow-up period of 42 weeks [24]. In addition, a study by Grotenhuis et al. showed decreased global systolic function detected by magnetic resonance imaging 16 years after an arterial switch operation [25]. This suggests that the current study’s findings may represent primary myocardial dysfunction that may later become more apparent by standard measurements of left ventricular function.

### Limitations

The present study is limited to a single center and a relatively small sample size for each cardiac anomaly. Hence, the findings obtained cannot be extrapolated to the broader population. This caveat necessitates caution in interpreting the results and precludes generalization of the findings. Moreover, our study could not provide long-term follow-up of longitudinal strain data to assess the course of a specific disease. The study’s design did not allow for accurate evaluation of disease progression due to the inclusion of patients at various age groups and postoperative intervals. The different follow-up intervals and the limited number of subjects might also affect the correlation between the strain values and operative and postoperative parameters. These limitations underscore the need for future research to employ a more focused cohort selection strategy. Finally, it should be noted that the current study did not compare the postoperative data with preoperative data. Instead, relied on the use of published normal values. Nevertheless, the findings demonstrate a similarity to those of earlier studies that have examined comparable healthy controls.

## Conclusion

Surgically repaired congenital heart disease patients exhibit abnormal global left ventricular longitudinal strain despite having normal left ventricle ejection fraction by conventional echocardiography. The study suggests that there is some impairment of left ventricular systolic function, which can be identified early in patients who have undergone cardiac surgery using two-dimensional speckle tracking echocardiography. The application of this method might help to guide the clinical management and longitudinal follow-up of patients after cardiac surgery such as early detection and management of heart failure.

## Data Availability

The datasets used and/or analyzed during the current study are available from the corresponding author on reasonable request.

## Abbreviations

2DSTE: Two-dimensional speckle tracking echocardiography
ASD: Atrial septal defect
AVSD: Atrioventricular septal defect
EF: Ejection fraction
LV: Left ventricle
TGA: Transposition of great arteries
TOF: Tetralogy of fallot
VSD: Ventricular septal defect
